# Viscous Fingering of Miscible Liquids in Porous and Swellable Media for Rapid Diagnostic Tests

**DOI:** 10.3390/bioengineering5040094

**Published:** 2018-10-29

**Authors:** Holly Clingan, Devon Rusk, Kathryn Smith, Antonio A. Garcia

**Affiliations:** 1 School of Molecular Sciences, Arizona State University, Tempe, AZ 85287, USA; 2 School of Biological & Health Systems Engineering, Arizona State University, Tempe, AZ 85287, USA; devon.rusk@asu.edu (D.R.); kcsmit20@asu.edu (K.S.); tony.garcia@asu.edu (A.A.G.)

**Keywords:** korteweg stresses, viscous fingering, glycerol, saliva, urease, ammonia, salivary urea nitrogen, diagnostic test strip, hue, saturation, brightness (HSB), electronic nose

## Abstract

In lateral flow and colorimetric test strip diagnostics, the effects of capillary action and diffusion on speed and sensitivity have been well studied. However, another form of fluid motion can be generated due to stresses and instabilities generated in pores when two miscible liquids with different densities and viscosities come into contact. This study explored how a swellable test pad can be deployed for measuring urea in saliva by partially prefilling the pad with a miscible solution of greater viscosity and density. The resultant Korteweg stresses and viscous fingering patterns were analyzed using solutions with added food color through video analysis and image processing. Image analysis was simplified using the saturation channel after converting RGB image sequences to HSB. The kinetics of liquid mixing agreed with capillary displacement results for miscible liquids undergoing movement from Korteweg stresses. After capillary filling, there was significant movement of liquid due to these fluidic effects, which led to mixing of the saliva sample with an enzyme test solution. Owing to the simplicity and speed of this test method, urea can be analyzed with an electronic nose over a useful range for detecting salivary urea concentration for rapid and early detection of dehydration.

## 1. Introduction

Rapid diagnostic tests are of great interest as a means of generating results with medical significance in a timely fashion to commence a course of treatment as early as possible and improve patient outcomes. Diagnostic tests that can be performed at the point of care or by the patient are highly desired due to the lower infrastructure costs and the potential for wide usage by expanding availability in low resource settings. One of the many challenges facing the development of a wide variety of tests involves sample acquisition and the level of invasiveness of the biological fluid collection process. Of recent interest has been the possibility of designing meaningful tests that collect and analyze biological fluid samples other than blood. The main challenge with collecting sweat, tears, saliva, urine, or other fluid excreted from the body is that biomarkers are likely present at very low concentrations. Moreover, when using these biological fluids, quantitative or semi-quantitative tests can be difficult to perform by a patient or at the point of care due to the need for applying and/or mixing the sample in a controlled fashion with the reagents and materials needed for the analysis, which can include enzymes or other biopolymers used to transduce the signal into a “readout”.

One idea, that to our knowledge has not previously been described in the literature, is to capitalize on Korteweg stresses and viscous fingering, which are phenomena studied to understand droplet motion in a continuous media, for soil environmental remediation, and oil recovery using water injected wells. Korteweg stresses are due to high concentration differences between two fluids, which are miscible but maintain an interface due to slow mixing. Viscous fingering is the generation of instabilities in fluid flow patterns due to the contact of two fluids with different viscosities. In porous media, viscous fingering will dominate how a less viscous fluid moves through the media when there are pores that contain a more viscous fluid. The dynamics also depend upon whether the fluids are miscible, whereby the absence of surface tension leads to more intense fingering patterns [1,2], and the high gradient of concentration leads to Korteweg stresses [3]. Density differences between the fluids play a minor role when the fluids are immiscible but play a major role with miscible fluids [3,4].

While from a practical perspective it would, at first thought, be disadvantageous to use viscous liquids for a rapid diagnostic test, since mixing would require more energy and the rate of diffusion would be reduced. However, this work demonstrates that in fact there are significant advantages in using a viscous solvent, such as glycerol or glycerol/water solutions. First, the presence of glycerol has been demonstrated to improve enzyme stability [5] and enzymes are popular in diagnostics tests due to their specificity and ability to amplify signals very efficiently. In this work, we use the example of glycerol as an additive to maintain the activity of urease to measure urea concentration in saliva as a biomarker for dehydration. Secondly, by preloading a porous and swellable gauze pad with a 50% glycerol/water urease preparation we show that a 10-min enzymatic assay can be conducted on a simulated saliva standard because the viscosity and density of saliva is lower than the enzyme-containing liquid initially present in the pores. The enzymatic assay is also aided by having rapid capillary action into pores that were not penetrated by the viscous enzyme-filled liquid, followed by liquid motion driven by viscous fingering instabilities, and mixing via Korteweg stresses. During the viscous fingering phase there is steady mixing of the two liquids over a wide area of the pad allowing for the detection of ammonia, as a reaction product, through a nanoporous polytetrafluoroethylene (PTFE) membrane (see Figure 13). This method is particularly attractive as a means of detecting urea or other biomarkers because a relatively large biological fluid sample (0.3 mL) can be analyzed to ensure that a sufficient level of biomarker is secured for analysis. An added benefit to this method is that when using food grade glycerol and enzymes, saliva testing could be done by the patient using a simple tray or by directly taking a sample from the mouth, after rinsing, and by pooling unstimulated saliva using standard protocols. 

In this paper, video time-lapse studies, a prototype electronic nose, and a 3-D printed cartridge are used to generate data to explore viscous fingering utilizing a commercial, off-the-shelf cotton gauze pad. The video studies are aided using food color to track fluid flow and by image processing to better elucidate flow and mixing patterns. The electronic nose with a 3-D printed cartridge is used to illustrate the potential benefit for assaying urea in saliva.

There are three distinct dynamic processes that are relevant in exploring Korteweg stresses and viscous fingering for rapid diagnostics. One dynamic process that we explore using video stills and image interpretation of liquid movement involves the kinetics of capillary action, which is generally very rapid (within seconds). The second dynamic process involves the final pattern generation due to concentration-driven flow and viscous fingering in the gauze pad, which is much slower (over 10–15 min) and can be better imaged by positioning the gauze pad horizontally. Interestingly, the effect of Korteweg stresses are observed when the higher density and viscous fluid is used in combination with the saliva sample, but viscous fingering is only observed when that solution is added first. Finally, in the third type of dynamics aided by the electronic nose, time dependent voltage measurement is due to enzymatic reaction and includes fluid mixing and reaction. For a 50% glycerol solution e enzyme reaction is on a similar time-scale to the formation of instabilities due to viscous fingering. Since, to the best of our knowledge, this is the first study of its kind, we hope that the data and analysis provided here can help generate interest in other uses of viscous fingering for bioassays in general.

## 2. Materials and Methods

### 2.1. Visualization Studies

Solutions of 50–50% (*V*/*V*) glycerol/water with Tartrazine (yellow food color dye), an aqueous pH 7 buffer solution with Tartrazine, and a pH 7 buffer with Brilliant Blue FCF (a triarylmethane, blue food color dye) were prepared. Additionally, solutions of pure glycerol and pure water with Tartrazine were prepared. Squares with 15 mm sides were cut from a non-stick gauze pad (Walgreens, Deerfield, IL, USA). Using a USB camera (Dino-Lite, Torrance, CA, USA), the pads were first placed vertically with one edge touching an aliquot of 300 μL. The pads were then placed, on the same edge and in the same manner, with 300 μL of the second solution. A time-lapse video was taken every 10 or 20 s for 15 min to analyze the change in color of the pad as the two solutions mixed, and to analyze how the front between the blue and yellow solutions moved and changed. ImageJ software (NIH, Bethesda, MD, USA) was used to create stills, which were then adjusted to maximum contrast and brightness, and subtracted from the initial image to better visualize any changes in color. ImageJ was also used with video stills to convert the RGB to HSB format and calculate pixel saturation statistics. These steps were done to understand fluid behavior for mixing of the preloaded enzyme and the saliva sample.

### 2.2. Salivary Urea Testing

Urease from Jack Beans (Carolina Biological, Burlington, NC, USA) was dissolved in either pH 7, 0.1 M phosphate buffer aqueous solution or in a 50% glycerol and 50% phosphate buffered aqueous solution at pH 7 (0.1 M). Both 6.4 units/mL urease solutions were filtered (0.2 micron) and stored at 4 °C. Saliva was collected, pooled, and analyzed for urea using QuantiQuik Urea Test Strips (BioAssay Systems, Hayward, CA, USA). Saliva containing normal levels of urea was used to generate test solutions in the range of 23–84 mg/dL salivary urea nitrogen by spiking concentrated urea (Sigma Aldrich, Inc., St. Louis, MO, USA). For each analysis, an aliquot of 300 microliters of urease solution was applied to a gauze pad in a 3-D printed cartridge using capillary action prior to applying the saliva test sample. The urea analysis was conducted using an electronic nose by measuring an aliquot of 300 microliters of saliva test solution and applying it to the gauze pad using capillary action for 20 s.

**Figure 1:**
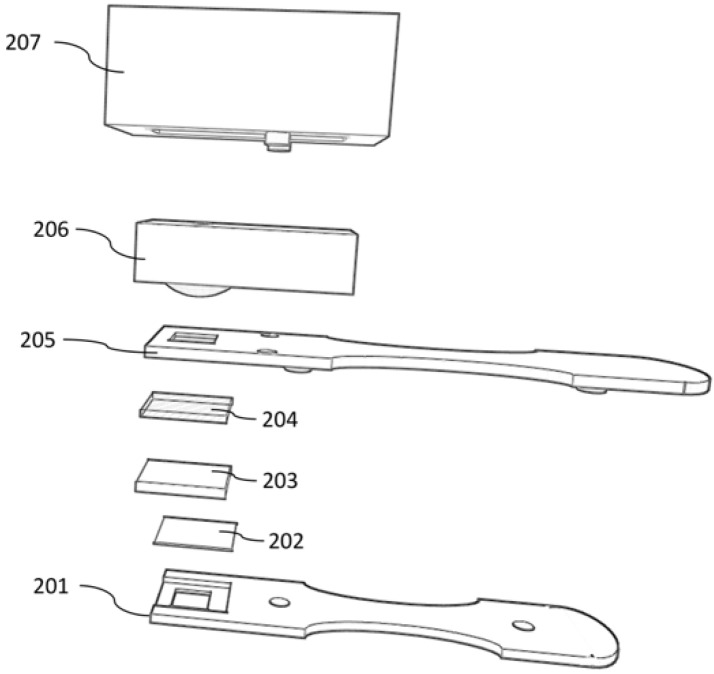
Schematic diagram of the electronic nose. 201 and 205—3-D printed housing; 202—plastic film; 203—gauze pad; 204—PTFE membrane; 206—MQ-135 sensor; and 207—3-D printed housing for electronic nose.

The electronic nose assembly was purposefully constructed using commercial, low cost components. A MQ-135 gas sensor (Zhengzhou Winsen Electronics Technology Co., Zhengzhou, China) was housed in a 3-D printed case designed to snap onto a 3-D printed test strip (Figure 1) on the side of the test strip containing a 0.22-micron PTFE membrane (Whatman, Maidstone, UK) window with dimensions comparable to the MQ-135 gas sensing outer mesh diameter. By connecting the MQ-135 to an Arduino Uno (Arduino, Somerville, MA, USA) and outputting the sensor voltage reading at a rate of 1 reading per second to a laptop, the sensor reading in air was read for 2–5 s before connecting to the test strip, followed by continuous reading for a maximum of 15 min. Prior to testing, ammonia solution standards from 0 to 1000 ppm were prepared and measured with the electronic nose assembly and compared with manufacturer’s supplied data on ammonia sensing to validate its performance.

The disposable test strip (Figure 1) was designed and printed to accommodate an electronic nose sensing attachment, as well as a colorimetric test strip. For this study, disposable test strips were fitted with a 15-mm square gauze pad, a clear plastic film, a 0.22-micron PTFE membrane, and a 5 × 10 mm velum plastic folded in half to wedge the gauze pad in the test strip and maintain good contact with the PTFE membrane.

## 3. Results

The following sections describe our experimental results designed to characterize and understand the role of capillary action, liquid mixing, Korteweg stress induced flow, and viscous fingering (see Figure 13) on a test pad consisting of a gauze pad with a preloaded enzyme solution, which is intended to measure the level of urea in saliva. Visualization results were described first, followed by testing of saliva with an electronic nose assembly.

### 3.1. Capillary Rise in a Gauze Pad

**Figure 2:**
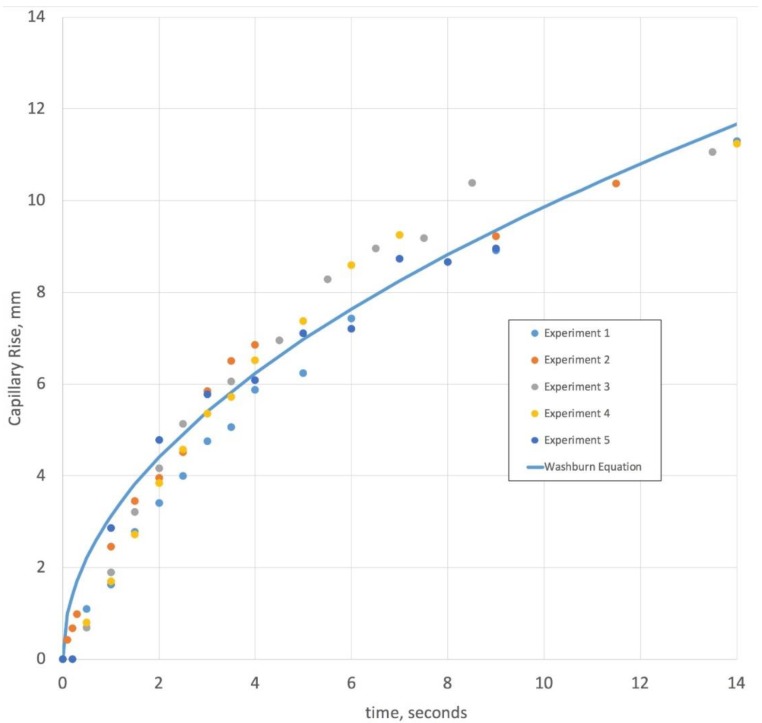
This figure shows the results of 5 capillary rise visualization experiments using 50% glycerol in water at 24 °C. The blue solid line is the best fit for the Washburn equation through the data points. The correlation coefficient is 0.98 for the 62 data points represented by filled circles of yellow, dark blue, light blue, orange, and grey.

As a means of verifying that time-lapse video and image processing of individual frames provide a useful means of tracking the liquid front, a 50% glycerol solution with yellow food coloring was videotaped while being imbibed by a section of gauze pad. Figure 2 shows the distance traveled versus time in 5 separate experiments. One useful way to understand the relationship between time and capillary level in these experiments is through Washburn’s Equation

(1) $$L^{2}=\frac{\gamma D\cos\phi }{4\eta }t,$$

where the capillary distance traveled, *L*, depends upon the liquid properties and the square root of time. While the gauze pad is a complex material due to not having fixed pores, and since it swells with liquid imbibition, a reasonable first approximation is the Washburn equation’s square root dependence on time. Additional terms with higher power dependence of time or a Darcy’s law pressure term can be used to account for pore resistance to flow changes upon swelling [6]. Another result worth noting is that the liquid level in the time-lapse video (see capillary action Supplemental video file) is not uniform throughout the pad. Because of this, the data was plotted using the liquid level in the middle range of the pad. Given this level variation, there is much less accuracy in the data during the first 2 s. Overall, the time for capillary filling is relatively rapid for a 50% glycerol aqueous solution, which has a viscosity about 8 times higher than water and nearly the same surface tension. The time scale of capillary filling for 100% water is much faster, suggesting that capillary pressure induced flow is a rapid process which is nearly completed within 10–20 s of contacting the pad with an aqueous solution between 0% and 50% glycerol.

### 3.2. Solution Mixing in a Capillary Tube

**Figure 3:**
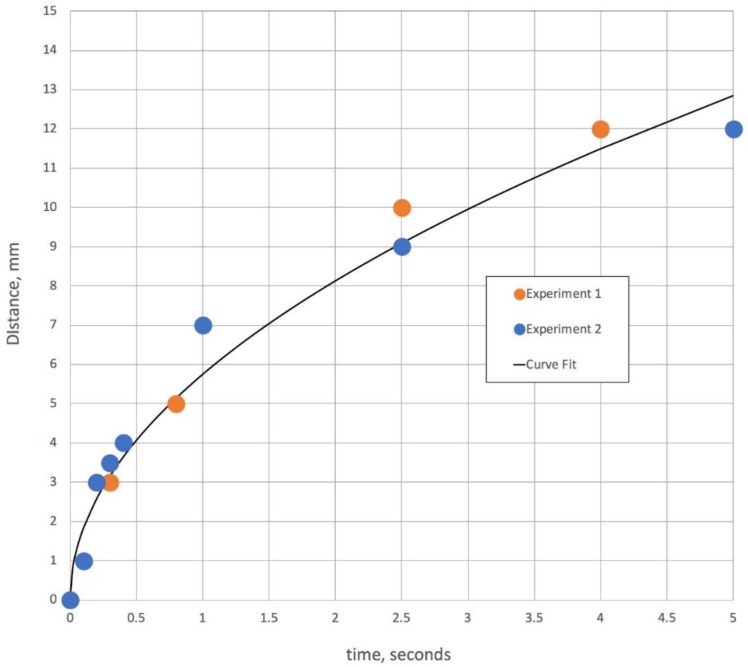
This figure is time versus distance of an aqueous solution front traveling from the capillary tube into a second capillary tube containing 50% glycerol. The curve fit is squared with distance and has a correlation coefficient of 0.98, which coincides with the equation for the water boundary given by Stevar and Vorobev [7] for miscible liquid/liquid interface movement in horizontal capillary tubes at initial mixing.

Another process that is relevant in understanding Korteweg stresses with two miscible solutions is the rate at which mixing can occur between two liquid fronts. It is also illustrative to use the same video time lapse system to observe mixing in two capillary tubes, where one tube contains water with a blue dye and the other a 50% glycerol solution with a yellow dye. Figure 3 gives the results of two video time-lapse movies showing that the aqueous solution penetrates the 50% glycerol capillary tube relatively far within a few seconds. For illustrative purposes and to compare these results with other investigations of water and glycerol dynamics in horizontal capillary tubes, a curve fit of *d* ∝ *t*^1/3^ is also shown in Figure 3. This exploration of mixing is intended to verify that in the porous network of the gauze pad, once the two liquid fronts meet, there is some degree of mixing in a very short timeframe. The interface between water and a 50% glycerol solution continues for longer periods of time, possibly for as long as 10,000 s [7].

### 3.3. Solution Mixing in a Gauze Pad

**Figure 4:**
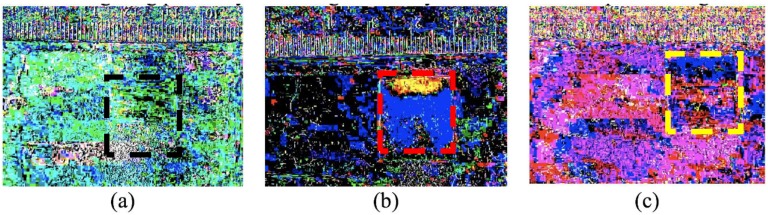
This figure shows the mixing of three combinations of two solutions. All images are the first frame in a 900-frame sequence, each subtracted from their respective final frame for a lapse-time of 15 min. The background and ruler are given for contrasting any changes that may occur in the porous swellable pad. The gauze pads are highlighted by dashed lines for easier visualization. The images are as follows: (**a**) Initially contains 100% water followed by 100% water, which exhibits no change in the pad; (**b**) Initially contains 50/50% glycerol followed by 100% water, which displays two lobes in blue and a region of yellow due to Korteweg stresses and viscous fingering; (**c**) Initially contains 100% glycerol followed by 100% water, which demonstrates that the initial 100% glycerol is too viscous to penetrate the pores in the pad.

From the data shown in the previous two sections, capillary rise and movement of the water front into a glycerol-rich phase can both occur very rapidly. In contrast, when applying two solutions to a gauze pad where the first solution is more viscous than the second, there can be kinetic processes in the scale of minutes for liquid movement due to pad swelling, viscous fingering, and Korteweg stresses. Moreover, there is an even slower process, in the time-scale of hours, for miscible liquid phase boundary smearing. Initial experiments when adding a more viscous and higher density liquid clearly indicated that viscous fingering instabilities are easily seen (Figure 4). After 15 min and subtracting the time lapse frame 1 from the final time-lapse frame (of the conditions indicated in Figure 4a–c), only when 50% glycerol is introduced first and then followed by water, is there a clear indication of where the water is dispersed through two lobes towards the bottom of the gauze pad (blue). The glycerol solution (yellow) is seen to predominate the top of the gauze pad, which is where both liquids were initially introduced.

**Figure 5:**
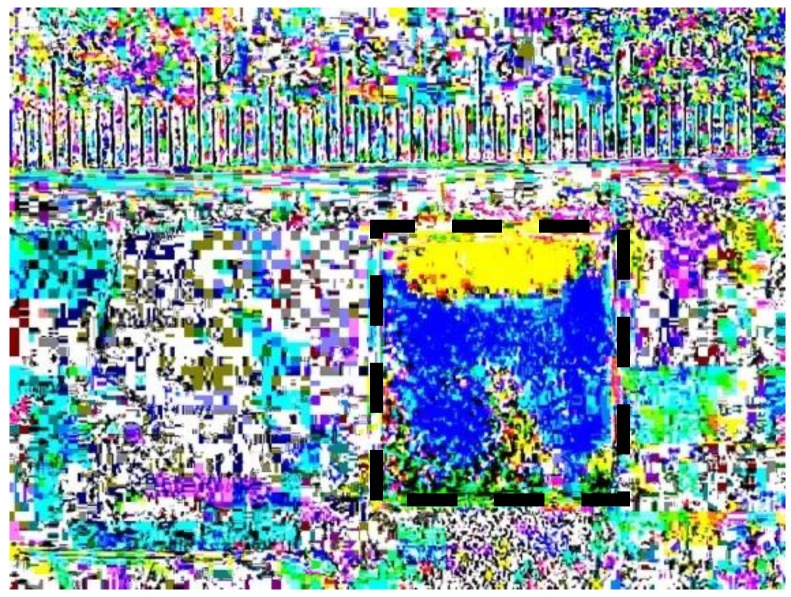
This figure displays an alternative to image subtraction, as seen Figure 4 panel (b). Here the first frame was inverted and was added to the final frame. This provides subtler details of color changes in the gauze pad (highlighted by dashed lines) by fluid movement. This allows for an alternate way to visualize the 50/50% glycerol followed by 100% water mixing combination, where two distinct lobes of water can be seen in blue and a yellow region of glycerol, which is caused by Korteweg stresses and viscous fingering.

Better definition of the flow pattern after 15 min is obtained when, as suggested by Knox et al. [8], the first image is inverted and then added to the last frame of the time-lapse movie (Figure 5). There appears to be phase mixing at the bottom of the gauze pad (green color) and in regions between the yellow and blue contours (green color). 

**Figure 6:**
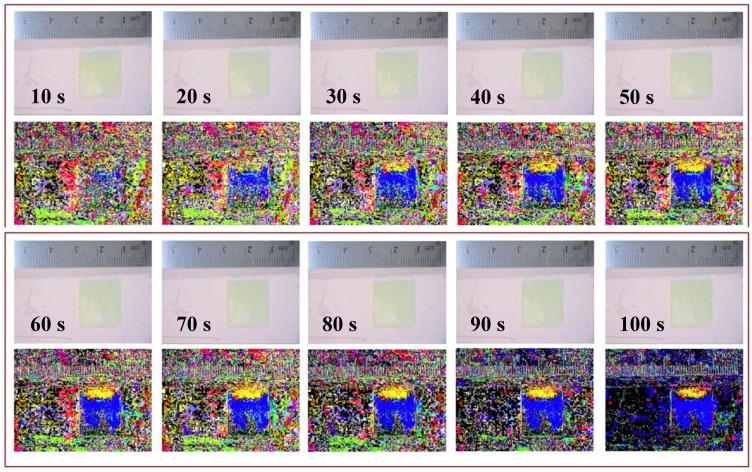
This figure shows 10 sets of 2 images, where the top image is the unprocessed still frame that includes a time stamp, and the bottom image is the result of subtracting that frame from the initial frame at time zero. This time-lapse comparison of subtracted images to unprocessed images is the series of frame at time zero. This time-lapse comparison of subtracted images to unprocessed images is the series of frames where solution (1) is 50% glycerol (yellow) and solution (2) is 100% water (blue).

Twenty-seven-time lapse videos, representing triplicates of the nine-possible combination for liquid 1 and liquid 2 for concentration levels of 0, 50, and 100% glycerol were taken (see Supplemental videos 1a–1c, 2a–2c, and 3a–3c, respectively, for representations). Figure 6 illustrates how the final pattern shown in Figure 4b appears 15 min after applying liquid 1 (50% glycerol) on the top end of the gauze pad for 20 s, followed by a 20 s gauze pad contact in the same location with liquid 2 (water).

**Figure 7:**
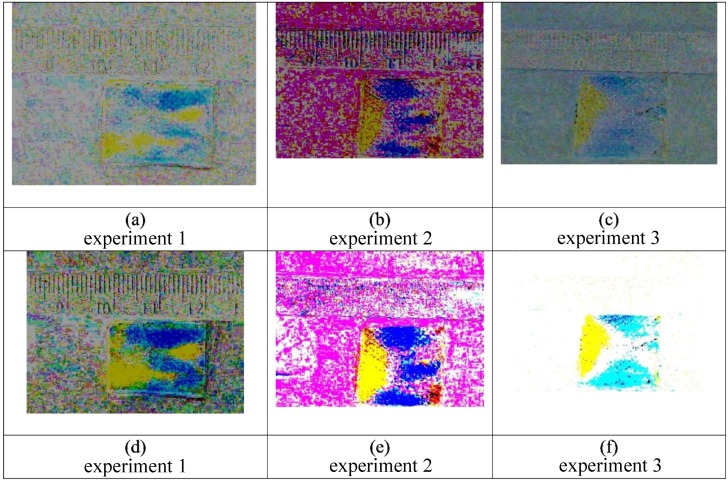
Triplicate experiments (1–3) are shown in the panels above for initial application of 50% glycerol (yellow) followed by 100% water (blue). The first frame is inverted and added to the final frame for each of the three separate experiments with the following conditions: Images (**a**–**c**) show the pattern after 5 min. The patterns remain, in large part, consistent after 15 min, as shown in images (**d**–**f**). The overall fraction of area of the fingering front (~0.6) is consistent with literature data and theory [4]. For frames (**d**–**f**) the yellow region is relatively consistent, while the blue regions vary more depending upon the propagation of the viscous fingering instabilities.

Indicative of the dynamic instability resulting in viscous fingering, the pattern of the phases for liquid 1 (50% glycerol) followed by liquid 2 (water) are not identical (Figure 7), but the fraction of area covered by the water front and the location of the glycerol phase are consistent throughout these replicate experiments.

**Figure 8:**
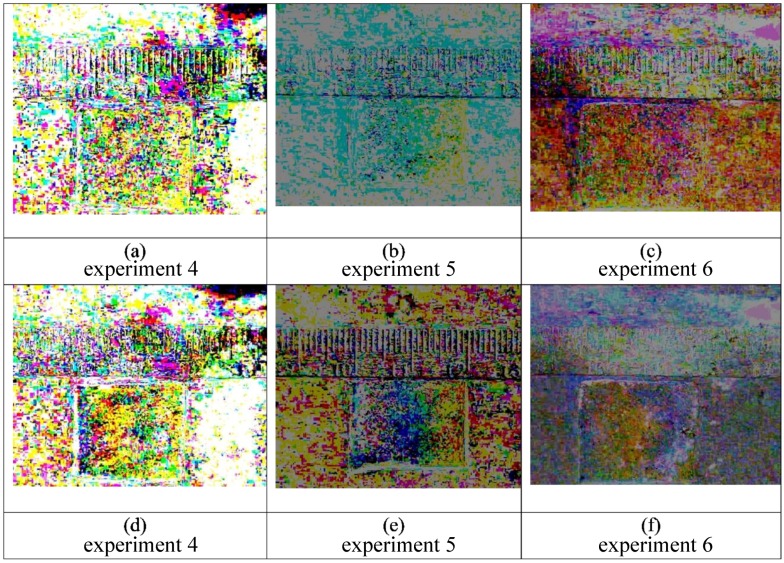
Triplicate experiments (4–6) are shown in the panels above for initial application of 100% water with yellow dye, followed by 100% water with blue dye. The first frame is subtracted from the final frame for each of the three separate experiments with the following conditions: Images (**a**–**c**) do not show distinct color variations in the pad after 5 min. Similarly, after 15 min there is no noticeable pattern in images (**d**–**f**).

**Figure 9:**
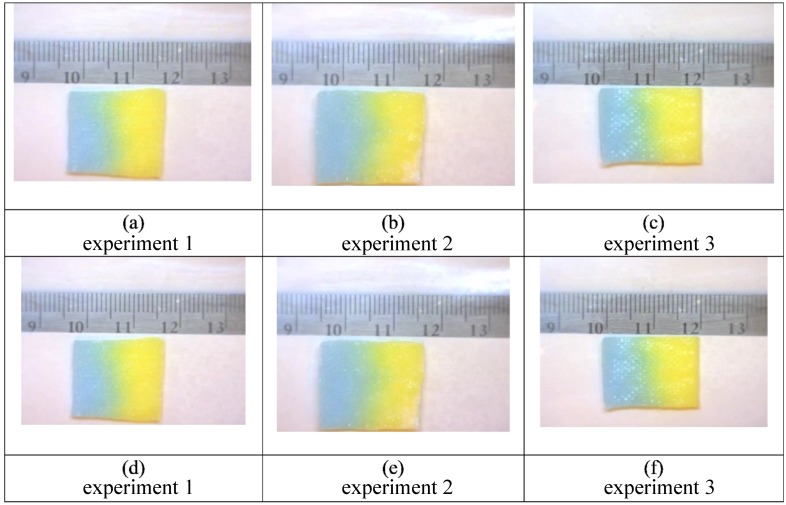
These are still frames from triplicate experiments (1–3) also shown in Figure 7. The panels above show unprocessed images for an initial application of 50% glycerol (yellow) followed by 100% water (blue). Images (**a**–**c**) are at 5 min and images (**d**–**f**) are at fifteen minutes. While there is some visual evidence of dye diffusion at the interface between the two colored liquids, the color variations within the gauze pads are not visible, as in Figure 7, without imaging processing to accentuate the subtle changes of color due to mixing.

When using the other 8 combinations of liquid 1 and liquid 2, there is no evidence of the overall pattern seen in Figure 7, which is seen to be well-formed within 5 min and continues to develop and stay consistent for 15 min. This result is congruent with the fact that two liquids of different viscosities and densities, where the high viscosity liquid is applied first, is the combination needed for viscous fingering. We also note in the Supplementary section videos 3a–3c that 100% glycerol is too viscous to penetrate the pores of the gauze pad to the extent found for water and for a 50% glycerol solution. Figure 8 and Figure 9 shows that there does not appear to be any discernable pattern after 5 min when using water with yellow dye followed by water with blue dye. At 15 min, there do seem to be regions with distinct color of either blue or yellow, indicating that some diffusion of the dyes is taking place.

### 3.4. Tracking an Enzymatic Reaction

**Figure 10:**
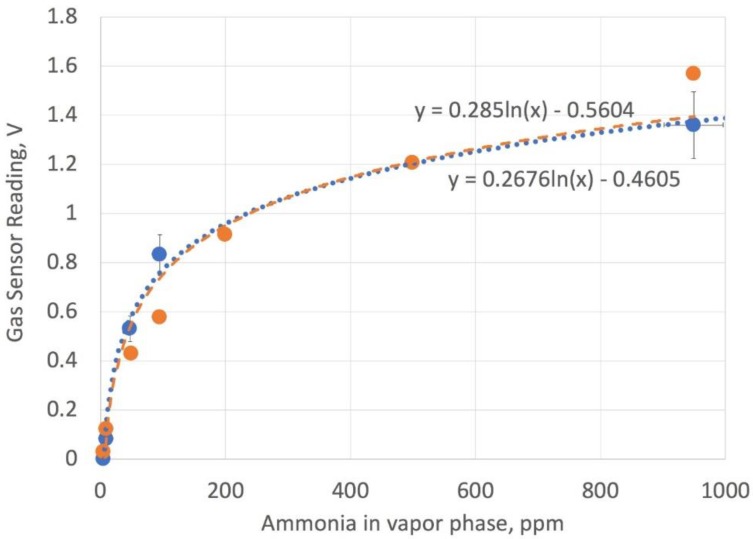
Calibration readings using the electronic nose assembly with ammonia solutions (blue filled circles and dashed line), compared to manufacturer’s data (orange filled circles and orange dashed line). The load resistor is set at 2.5 k Ohms.

To test whether the electronic nose assembly can accurately track ammonia evolved from an enzymatic reaction of urea, ammonia solutions were prepared and introduced into the gauze pad by capillary action. Figure 10 shows a comparison of the calibration data based on converting ammonia solution phase concentration to vapor phase using Henry’s law and the manufacturer’s data. The gas sensor had an adjustable load resistor, and to have better linearity at low concentration, the resistor was set to 2.5 k-Ohms. The sensor readings were taken over 15 min and the maximum voltage values averaged after 5 min were found to yield consistent results. The values were relatively constant from 5–15 min, except at the higher values of ammonia, where values drift downward, presumably due to the slow leaking of ammonia gas from the reading chamber. The need to wait for about 5 min to get an accurate value is attributed to the time needed to accumulate a vapor phase of ammonia in the sensor chamber. Fitting the data to an exponential curve gave a reasonable agreement between the electronic nose assembly values and the best fit of the manufacturer’s data.

**Figure 11:**
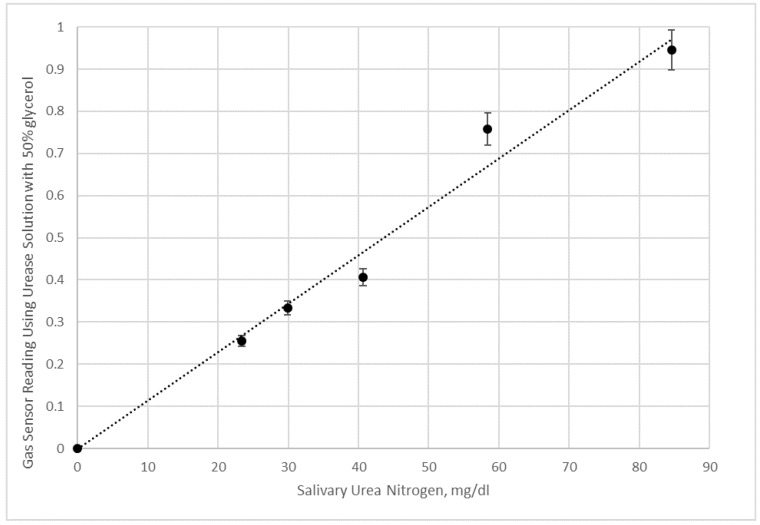
Here electronic nose measurements of saliva samples and saliva samples with added urea given in terms of salivary urea nitrogen were done in duplication. Data shown are using a 50% glycerol urease solution.

**Figure 12:**
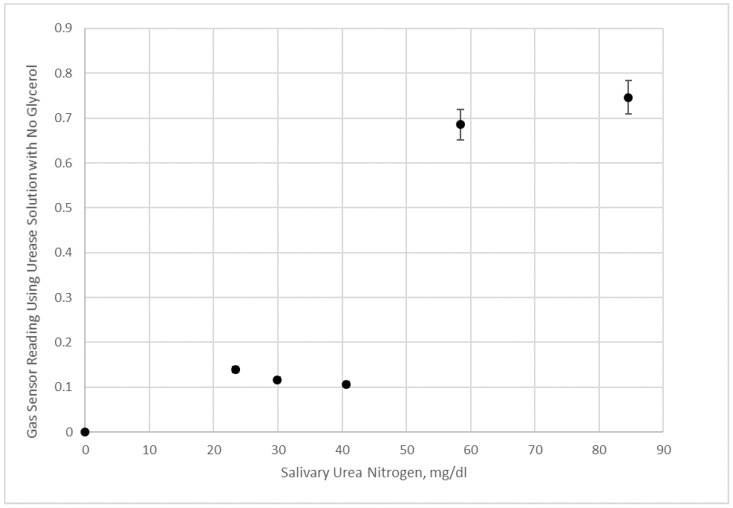
Electronic nose measurement of saliva samples and saliva samples with added urea given in terms of salivary urea nitrogen. Data shown are using a urease solution with no added glycerol.

Duplicate experiments of saliva samples and saliva samples with added urea were conducted using urease solutions with 50% glycerol (Figure 11) and no glycerol (Figure 12) in pH 7 aqueous buffer. Since the pH of the reaction solution is not adjusted to shift the equilibrium towards ammonia, and hence ammonium ion is present at a significant fraction, the sensor values in Figure 10 and Figure 11 are lower than what would be measured by raising the pH. Nonetheless, the data in Figure 10 for the 50% glycerol enzyme solution shows a good linear correlation with salivary urea nitrogen in the sample.

However, Figure 12 data with an aqueous urease solution does not appear to be sensitive enough at lower salivary urea nitrogen to distinguish small concentration differences. We interpret these results as agreeing with the visualization experiments, since substantial mixing using 50% glycerol urease test liquid occurs within minutes of applying the saliva sample to the test strip (which is attached to the electronic nose after 20 s of initial imbibition of the sample). This form of mixing generates the results in Figure 11 presumably by creating a higher surface area of contact for reactions, which should occur initially at the glycerol enzyme solution/saliva solution interface and spread as urea diffuses into the glycerol enzyme phase.

## 4. Discussion

### 4.1. Visualization Experiments

**Figure 13:**
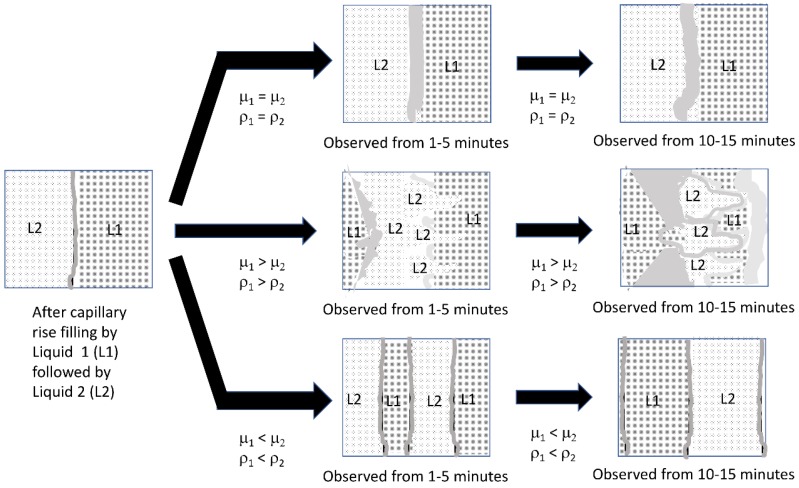
Characterization of liquid front movements in the visualization experiments, where (1) is the initial liquid loaded into the gauze pad via capillary action, and (2) is the second liquid applied via capillary action. Each liquid is added at 300 μL and the total capacity of the gauze pad for liquid is 600 μL.

The results of the gauze pad visualization after adding both liquids indicated that there were three categories of results, as illustrated in the schematic diagram in Figure 13. When the viscosity and density of the two liquids match (top arrow), there was some low level of mixing due to diffusion at the interface. However, when liquid 2 had a higher viscosity and density than liquid 1 (bottom arrow) and both liquids were miscible (see supplemental files), steady movement by both liquid fronts was observed resulting in a wide zone of mixed (green color) liquid. When the order of addition was reversed and the more viscous and higher density liquid was added first (middle arrow), liquid movement was observed leading to mixing, but the pattern was generally two lobes or “fingers”.

We attribute the movement due to the action of combining a 50% glycerol solution and a water solution in pores leading to Korteweg stresses

(2) $$T_{11}=k{\left(\frac{\partial C}{\partial x}\right)}^{2},$$

in the direction of action given by the above equation, the stress, *T*, is caused by the square of the gradient in concentration [9]. The symbol *k* is a nonnegative constant and the stress tensor would be added to the equation of motion as an additional pressure term in the form of a divergence. Relating to the condition of viscous fingering, the relationship posed by Saffman and Taylor [4],

(3) $$\left(\frac{\mu_{2}}{k_{2}}-\frac{\mu_{1}}{k_{1}}\right)V+\;\left(\rho_{2}-\rho_{1}\right)g<0,$$

predicts instabilities when the first liquid (subscript 1) has higher density and viscosity than the second liquid (subscript 2). Since this condition is met by the order of 50% glycerol solution followed by a water solution, as shown in the middle arrow of Figure 13, and not when the solution properties of the bottom arrow are present, we attribute the complex patterns we observe in our visualization experiments as due to viscous fingering.

**Figure 14:**
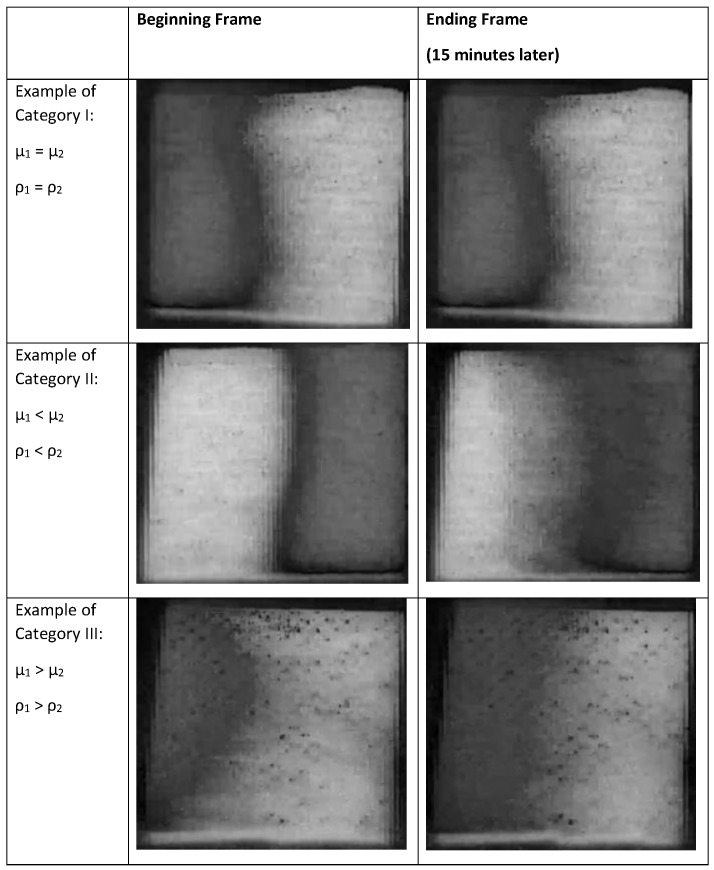
Saturation values of the first and final frame (after 15 min) using the saturation component of the HSB image after converting from RGB in ImageJ. The three categories of fluid filling are based on having two miscible fluids with the same or different values of viscosity and density.

A study of 100% glycerol placed in a capillary tube, which is subsequently immersed at both ends into reservoirs of water [7], showed that the interface between the two liquids are visible even after 10,000 s and the interface moves with a 2/3 (short time period) and 1/3 power relationship (overall), with respect to time. For our study, it was difficult to determine the speed of movement using RGB images of the gauze pad. A better method to gauge changes was to convert the RGB images to the hue, saturation, brightness format (HSB), and measure the saturation values of the mean pixel value and the pixel value distributions, which are given in a scale of 0–255. By studying videos of a yellow and a blue drop on a water repellant surface converging to form the color green, the saturation pixel values were determined to be in the order of yellow > blue > green. This order remained consistent with the gauze pad images and the saturation levels are distinct enough to visually track the changes, as seen in Figure 14.

**Figure 15:**
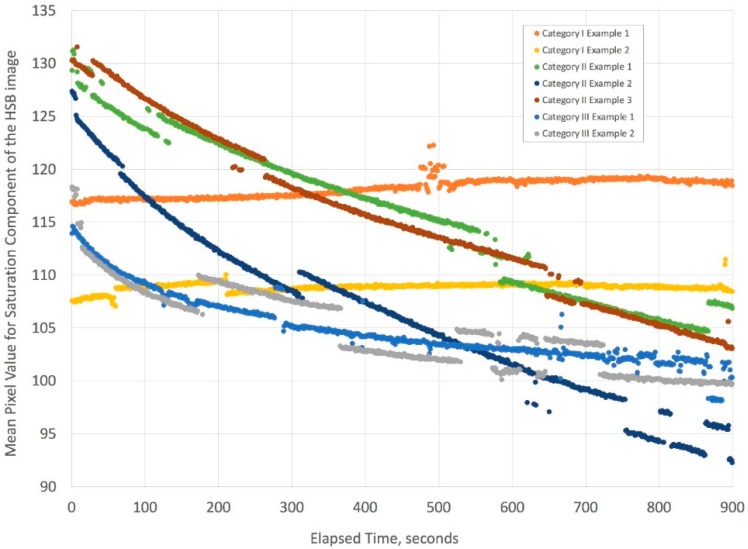
This complex figure illustrates how the mean pixel value changes with time. The categories in the legend refer to the physical properties of the liquids described in Figure 13. The two separate experiments for Category I show no variation in mean pixel value in time because the liquids have the same density and viscosity. The three separate experiments for Category II, which are for liquid conditions where Korteweg stresses and viscous fingering can occur, show a steep decline in mean pixel value over time. Finally, the two separate experiments for Category III, where liquid conditions generate Korteweg stresses, show a shallower decline in the mean pixel value over time. Owing to slight variations in lighting for short times during the time-lapse videos, there are minor discontinuities in the mean pixel values, but the curves retain similar slopes. Overall, the trend for Category I (duplicate) is essentially that there is no substantial mixing of the liquids, while the decline in mean pixel values seen for Categories II (triplicate) and III (duplicate) are the result of decreases in pixel saturation due to mixing of the yellow and blue color liquids to yield the color green (see Supplemental File).

A histogram analysis of these and the drop saturation component of the HSB images is provided in the supplemental file section, but a more compact representation of the dynamics of mixing is given by analyzing the mean pixel values over time for the three categories of gauze filling (see Figure 15). The data clearly indicates that Korteweg stresses with or without viscous fingering create substantial mixing, thereby lowering the saturation values of the gauze image steadily during the 15 min of imaging. When the two liquids have the same density and viscosity (Category I), Figure 15 shows a slight increase in mean pixel value. We interpret this result as indicating essentially no significant mixing over 15 min.

A brief analysis of the Category II profile using the saturation values indicates that the liquid fronts move in a power law relationship very similar to what the literature [7,10] indicates, namely within the range of about ½ power in time. More careful experimentation using a better-controlled interface is recommended for a more exact comparison to the literature, but this is outside the scope of our study. For now, our data indicated that a 50% glycerol solution when combined with an aqueous sample travels through a gauze pad based on similar dynamics suggested by capillary studies.

### 4.2. Implications for Rapid Testing

Rapid testing of urea can be performed via colorimetric test strips, such as the one described in the experimental section (QuantiQuik Urea Test Strip), and several different test strip formats are described in the literature. The test system described here and in the other test strips are based on urease catalysis of urea to ammonia and carbon dioxide.

$$\;{\mathrm{CH}}_{4}N_{2}0\;+{\;H}_{2}0\;\to \;2{\mathrm{NH}}_{3}\;+{\;\mathrm{CO}}_{2}\;$$

However, the test strips are semi-quantitative and based on an indirect measurement of ammonia due to the need for a chromogenic readout using a pH indicator, which is also less sensitive than directly measuring ammonia. They also require a precise amount of sample (usually 20 microliters) due to the need for hydration and mixing of a small amount of urease enzyme dried on the test strip. Our lab is interested in having a more quantitative system to reproducibly probe the range between normal of <20 mg/dL urea nitrogen and high >40 mg/dL urea nitrogen to more quickly track dehydration.

Since the clinical literature indicates that salivary and blood urea nitrogen track reasonably well [11], our goal was to use more quantitative and direct detection of ammonia to improve interventions aimed at averting dehydration for patients with chronic illnesses who have this condition as a side effect, or in situations where dehydration is occurring over a span of hours or days due to intense physical exertion or lack of proper hydration care. 

In seeking a method with the potential for quantitation of urea in the desired range and a disposable test strip that would not need precise saliva sample measurement, it became apparent that an approach based on passive microfluidics would have the best possibility to meet the stated needs. Another consideration was to have a test that could yield results relatively rapidly in the time scale that lateral flow tests strips provide readings, namely in about 5–15 min. Since glycerol added to enzyme solutions is very useful to maintain enzyme activity, we experimented with adding up to 50% glycerol when preparing a urease solution and adding it to a cotton gauze pad. We corroborated literature reports that urease can be stored in glycerol solutions and on a non-protein adherent gauze pad (in a sealed container) at 4 °C for 8 months. From a practical perspective, we also determined that the glycerol/enzyme solution penetrated the gauze pad to a self-limiting level using capillary action.

Initially, we believed that while glycerol was a useful additive to preserve enzyme, and potentially contacting with the enzyme in solution form would yield faster kinetics than trapping enzyme in the pores, but diffusion of urea was thought to be a potential problem. It was only after we conducted simple visualization studies that we were surprised by how the saliva sample would rapidly move within the gauze pad after it was imbibed by capillary action. Afterwards, using a gas sensor as an electronic nose, we realized that Korteweg stresses and viscous fingering were playing important roles in measuring the level of ammonia product. It would seem reasonable to assume that most of the detection of ammonia in Figure 11 is due to enzymatic reaction at the interface between the phases throughout the gauze pad, whilst in Figure 12 the reaction is mostly occurring in the center with some mixing due to the initial application of saliva contacting the enzyme solution, and in addition, due to some across the liquid fronts.

Since the research described here was an initial study of Korteweg stresses and viscous fingering for diagnostics, it is important to note that a range of glycerol concentration and the use of different polyols or equivalent compounds of similar enzyme stabilization and viscosity enhancement could be tested to optimize results for urea measurement or detection of other biomarkers using enzymes.

## 5. Conclusions

Salivary urea can be measured at the point of care using a rapid colorimetric test strip or by collecting a sample for processing in a clinical laboratory facility. Both measurement methods rely on enzymatic reaction using urease to determine the urea nitrogen concentration in the standard clinical units of mg/dL. The rapid colorimetric test strip has the advantages of convenience and low cost; however, the accuracy is limited due to reliance on distinguishing between shades of green above urea nitrogen of 15 mg/dL. This is problematic, especially between 25 and 55 mg/dL, which is a range of interest that encompasses a transition from mild to high dehydration. Moreover, the color change is an indirect measure of urea reaction to the product ammonia, since it reflects a change in the pH of the test sample, which may vary due to a variety of underlying oral and digestive health conditions. The clinical laboratory urea assay kits also use the enzyme urease, but these microplate assays use a chromogen that reacts directly with ammonia. The resultant product optical density between 580 nm and 630 nm is then measured using a spectrophotometer, yielding a very linear and quantitative reading for urea nitrogen concentrations up to 45 mg/dL. Dilutions, urea standards, and control microwells (without addition of urease) gives clinical laboratory urea assays correlation coefficients usually greater than 0.99, even in the very low range of urea nitrogen.

Subtle liquid motion that is difficult to detect, especially when mixing different liquids, can be tracked and analyzed using dyes that are soluble in the test liquids. Our studies show that Korteweg stresses and instabilities leading to viscous fingering [12] with miscible liquids of different viscosity are responsible for significant motion of the test sample through the porous media, as this motion is only observed when there are suitable viscosity and density gradients between the two fluids. Testing via passive microfluidics utilizing density and viscosity gradients for urea detection in saliva, holds promise as a simple way to quantitate salivary urea nitrogen in the range of interest for normal and slightly elevated levels of urea via a simple gas sensor integrated with a gauze pad and a PTFE membrane to create an electronic nose assembly. Converting RGB images into HSB images and tracking the saturation component yielded more information on how the properties of the liquids and the order in which they are imbibed by the gauze pad generate different mixing patterns. To create substantial mixing over the test pad, imbibing a liquid of higher viscosity and density than the biological liquid sample is preferred. Given that glycerol and similar compounds can stabilize enzymes and are generally compatible with biological fluids, are miscible with water, and have the requisite physical properties, there could be many assays performed using this format.

## Supplementary Materials

Supplementary materials are available online at http://www.mdpi.com/2306-5354/5/4/94/s1. Supplemental file: Analysis of Saturation Component for HSB Images: Figure S1: Visualization of Two Drops Combining on a Hydrophobic Surface; Figure S2: Histogram of Saturation Component Pixel Levels; Figure S3: Histograms of the Saturation Component for the First Frame; Figure S4: Histograms of the Saturation Component for the Last Frame.
