# Supplementary material for: Viscous Fingering of Miscible Liquids in Porous and Swellable Media for Rapid Diagnostic Tests
Source: Bioengineering (Basel). 2018 Oct 29;5(4):94. doi: 10.3390/bioengineering5040094 (PMC6315716; doi:10.3390/bioengineering5040094)
Supplement: Supplementary file 1 [file bioengineering-05-00094-s001.pdf]

Analysis of Saturation Component for HSB Images

Viscous Fingering of Miscible liquids in Porous and Swellable Media for Rapid Diagnostic Tests

Holly Clingan, Devon Rusk, Kathryn Smith, and Antonio A. Garcia

In order to better understand mixing within the gauze pad, the RGB video stills from the 10 videos uploaded to this supplemental file section were converted to HSB format and the saturation component was analyzed using ImageJ to calculate the mean pixel value over time as well as the distribution of pixels. The mean pixel value information over the pad with time was directly calculated from ImageJ. However, the distribution of pixels which generates a histogram plot in ImageJ requires more analysis in order to interpret the relative proportion of yellow, blue, and green liquid which can better indicate the degree of mixing.

The basis used to determine the three distinct distribution of pixels for the saturation component of the HSB files for yellow, blue, and green colored liquid is based on a visualization of two drops combining on a hydrophobic surface. The simple experiment to establish the histograms is visualized using three stills from one movie where the yellow colored drop is placed near the blue colored drop and one drop is increased until the drops merge to form a green drop.

Figure S1. Visualization of Two Drops Combining on a Hydrophobic Surface

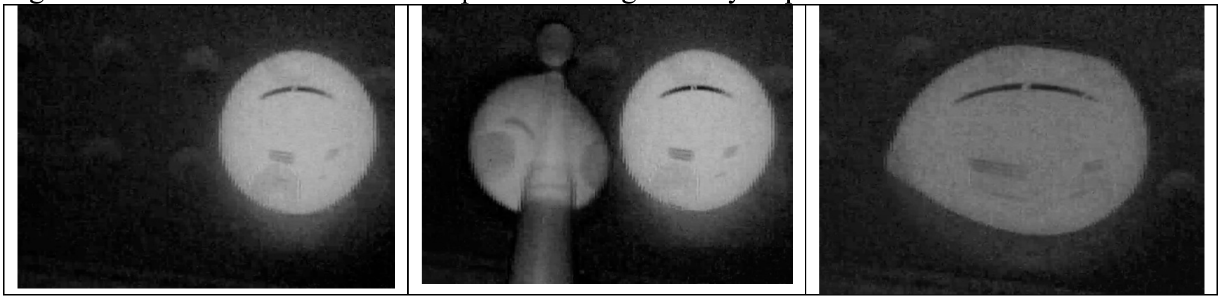

The resultant histograms of the saturation component pixel levels were fitted to Gaussian distributions, giving the following fit and components

Figure S2. Histograms of the Saturation Component Pixel Levels

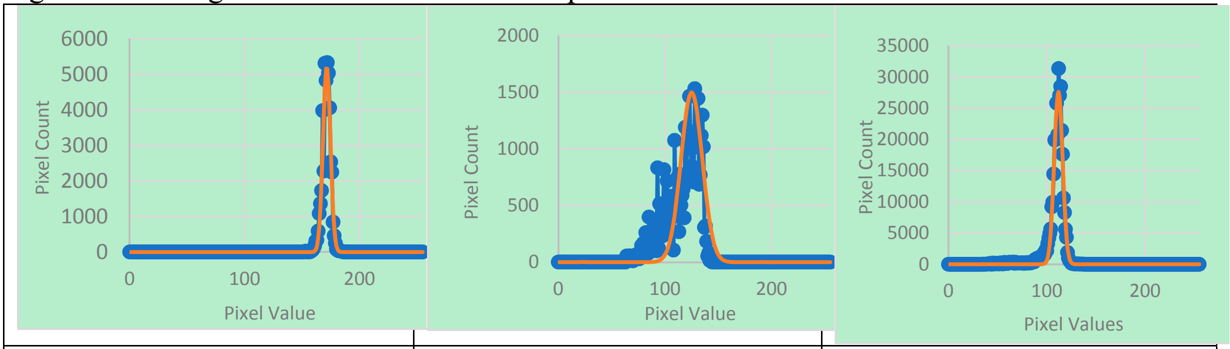

Yellow<br>Mean = 171<br>Amplitude = 5,200<br>Width @ inflection = 21	*Blue*<br>Mean = 125<br>Amplitude = 1,500<br>Width @ inflection = 200	*Green*<br>Mean = 112<br>Amplitude = 28,000<br>Width @ inflection = 37

where it is apparent that yellow > blue > green with blue being relatively close to the mean saturation value of the green color resulting from the combined drops. Since saturation relates to the greyness of the color, the order of pixel values is reasonable since color charts containing yellow, blue, and green colors show a similar trend.

When applying this approach to the gauze pad, we noted that due to reflection of the surface and the mixed image of the cotton fibers the saturation levels are not the same. However, the trend remained yellow > blue > green, so we approached the analysis as three gaussian distributions in saturation due to the presence of all three colors to a lesser extent in the first frame and, if mixing is substantial, higher levels of green in the final frame taken 15 minutes later.

Here are representative histograms for the three Liquid 1 and Liquid 2 combinations by processing the 9 videos uploaded into the supplemental section.

The corresponding video files that visualize these conditions can be found at https://repository.asu.edu/items/50334. As indicated in Figures S3 and S4, Videos 1a – 1c represent 100% water with blue dye and 100% water with yellow dye; videos 2a – 2c contain solutions of 100% water with yellow dye and 50% glycerol with blue dye; videos 3a – 3c contains 50% glycerol with yellow dye and 100% water with blue dye.

Figure S3. Histograms of the Saturation Component for the First Frame

<table>
<tr><td>Liquid 1: water
Liquid 2: water
Videos: 1a – 1c</td><td>Liquid 1: water
Liquid 2: 50% glycerol
Videos: 2a – 2c</td><td>Liquid 1: 50% glycerol
Liquid 2: water
Videos: 3a – 3c</td></tr>
<tr><td>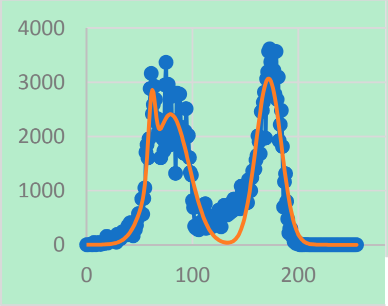
</td><td>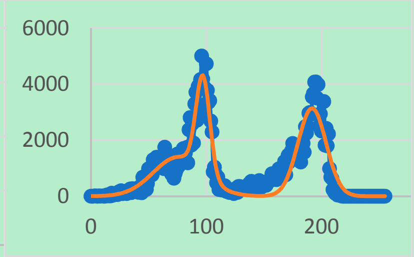
</td><td>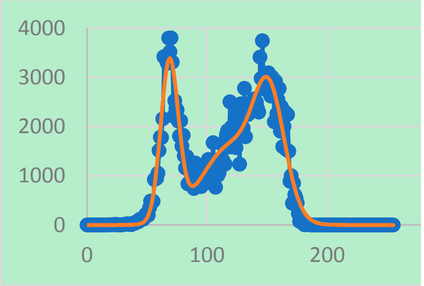
</td></tr>
<tr><td>Yellow
Mean = 172
Amplitude = 3,000
Width @ inflection = 284

Blue
Mean = 79
Amplitude = 2,400
Width @ inflection = 620

Green
Mean = 61</td><td>Yellow
Mean = 193
Amplitude = 4,000
Width @ inflection = 250

Blue
Mean = 97
Amplitude = 5,000
Width @ inflection = 76

Green
Mean = 60</td><td>Yellow
Mean = 152
Amplitude = 2,200
Width @ inflection = 330

Blue
Mean = 120
Amplitude = 1,600
Width @ inflection = 1.260

Green
Mean = 69</td></tr>
</table>

Amplitude = 21<br>Width @ inflection = 1,400	Amplitude = 1,300<br>Width @ inflection = 30	Amplitude = 3,200<br>Width @ inflection = 110

Figure S4. Histograms of the Saturation Component for the Last Frame

<table>
<tr><td>Liquid 1: water
Liquid 2: water
Videos: 1a – 1c</td><td>Liquid 1: water
Liquid 2: 50% glycerol
Videos: 2a – 2c</td><td>Liquid 1: 50% glycerol
Liquid 2: water
Videos: 3a – 3c</td></tr>
<tr><td>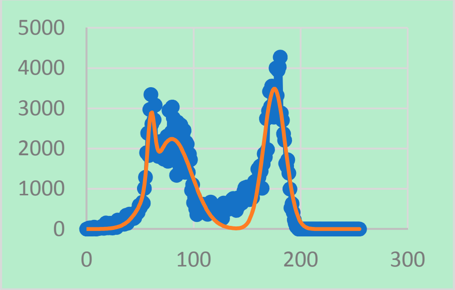
</td><td>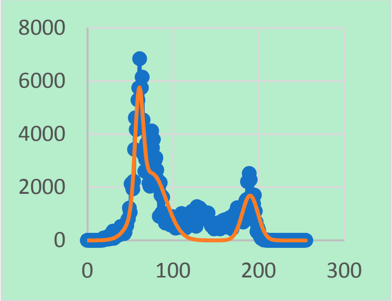
</td><td>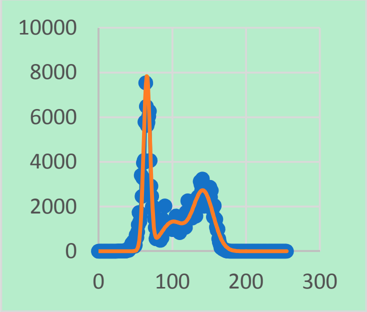
</td></tr>
<tr><td>Yellow
Mean = 175
Amplitude = 3,500
Width @ inflection = 200

Blue
Mean = 80
Amplitude = 2,200
Width @ inflection = 690

Green
Mean = 60
Amplitude = 21
Width @ inflection = 1,601</td><td>Yellow
Mean = 190
Amplitude = 2,500
Width @ inflection = 50

Blue
Mean = 97
Amplitude = 1,000
Width @ inflection = 50

Green
Mean = 60
Amplitude = 30
Width @ inflection = 7,000</td><td>Yellow
Mean = 143
Amplitude = 2,400
Width @ inflection = 150

Blue
Mean = 120
Amplitude = 1,200
Width @ inflection = 150

Green
Mean = 65
Amplitude = 7,800
Width @ inflection = 46</td></tr>
</table>
